# Gut microbiota dysbiosis is associated with altered tryptophan metabolism and dysregulated inflammatory response in COVID-19

**DOI:** 10.1038/s41522-024-00538-0

**Published:** 2024-08-01

**Authors:** Morgan Essex, Belén Millet Pascual-Leone, Ulrike Löber, Mathias Kuhring, Bowen Zhang, Ulrike Brüning, Raphaela Fritsche-Guenther, Marta Krzanowski, Facundo Fiocca Vernengo, Sophia Brumhard, Ivo Röwekamp, Agata Anna Bielecka, Till Robin Lesker, Emanuel Wyler, Markus Landthaler, Andrej Mantei, Christian Meisel, Sandra Caesar, Charlotte Thibeault, Victor M. Corman, Lajos Marko, Norbert Suttorp, Till Strowig, Florian Kurth, Leif E. Sander, Yang Li, Jennifer A. Kirwan, Sofia K. Forslund, Bastian Opitz

**Affiliations:** 1 Experimental and Clinical Research Center (ECRC), a cooperation of the Max Delbrück Center and Charité–Universitätsmedizin, Berlin, Germany; 2 Max Delbrück Center for Molecular Medicine in the Helmholtz Association (MDC), Berlin, Germany; 3 Charité–Universitätsmedizin Berlin, a corporate member of Freie Universität Berlin and Humboldt-Universität zu Berlin, Berlin, Germany; 4 Department of Infectious Diseases, Respiratory Medicine and Critical Care, Charité–Universitätsmedizin Berlin, a corporate member of Freie Universität Berlin and Humboldt-Universität zu Berlin, Berlin, Germany; 5 Berlin Institute of Health (BIH) at Charité, BIH Metabolomics Platform, Berlin, Germany; 6 Berlin Institute of Health (BIH) at Charité, Core Unit Bioinformatics, Berlin, Germany; 7 Department of Computational Biology for Individualized Infection Medicine, Center for Individualized Infection Medicine (CiiM), a joint venture between the Helmholtz-Center for Infection Research (HZI) and the Hannover Medical School (MHH), Hannover, Germany; 8 TWINCORE, joint ventures between the Helmholtz Center for Infection Research (HZI) and the Hannover Medical School (MHH), Hannover, Germany; 9 College of Life Sciences, Beijing Normal University, Beijing, China; 10 Department of Microbial Immune Regulation, Helmholtz Center for Infection Research (HZI), Braunschweig, Germany; 11 German Center for Infection Research (DZIF), partner site Hannover-Braunschweig, Braunschweig, Germany; 12 Berlin Institute for Medical Systems Biology, Max Delbrück Center for Molecular Medicine in the Helmholtz Association (MDC), Berlin, Germany; 13 Institute of Biology, Humboldt-Universität zu Berlin, Berlin, Germany; 14 Labor Berlin-Charité Vivantes GmbH, Berlin, Germany; 15 Institute of Medical Immunology, Charité–Universitätsmedizin Berlin, a corporate member of Freie Universität Berlin and Humboldt-Universität zu Berlin, Berlin, Germany; 16 Institute of Virology, Charité–Universitätsmedizin Berlin, a corporate member of Freie Universität Berlin and Humboldt-Universität zu Berlin, Berlin, Germany; 17 German Center for Infection Research (DZIF), Berlin, Germany; 18 German Center for Cardiovascular Research (DZHK), partner site Berlin, Berlin, Germany; 19 German Center for Lung Research (DZL), Berlin, Germany; 20 University of Nottingham School of Veterinary Medicine and Science, Loughborough, UK; 21 Structural and Computational Biology Unit, European Molecular Biology Laboratory (EMBL), Heidelberg, Germany

**Keywords:** Health care, Microbiome, SARS-CoV-2

## Abstract

The clinical course of COVID-19 is variable and often unpredictable. To test the hypothesis that disease progression and inflammatory responses associate with alterations in the microbiome and metabolome, we analyzed metagenome, metabolome, cytokine, and transcriptome profiles of repeated samples from hospitalized COVID-19 patients and uninfected controls, and leveraged clinical information and post-hoc confounder analysis. Severe COVID-19 was associated with a depletion of beneficial intestinal microbes, whereas oropharyngeal microbiota disturbance was mainly linked to antibiotic use. COVID-19 severity was also associated with enhanced plasma concentrations of kynurenine and reduced levels of several other tryptophan metabolites, lysophosphatidylcholines, and secondary bile acids. Moreover, reduced concentrations of various tryptophan metabolites were associated with depletion of *Faecalibacterium*, and tryptophan decrease and kynurenine increase were linked to enhanced production of inflammatory cytokines. Collectively, our study identifies correlated microbiome and metabolome alterations as a potential contributor to inflammatory dysregulation in severe COVID-19.

## Introduction

The Coronavirus disease 2019 (COVID-19) pandemic, caused by the severe acute respiratory syndrome coronavirus 2 (SARS-CoV-2), has affected over 700 million individuals and resulted in more than 7 million deaths worldwide by early March 2024 (https://data.who.int/dashboards/covid19/). The infection typically starts with mild to moderate respiratory symptoms. After approximately one week, a minority of infected individuals develop pneumonia which may be complicated by acute respiratory distress syndrome (ARDS), coagulopathy, and multiorgan failure^1,2^. The common kinetics of disease progression together with recent observational studies suggest that COVID-19 severity is primarily driven by a dysregulated, not adequate and often excessive immune response. Several studies found high levels of proinflammatory cytokines, such as interleukin (IL)-6, tumor necrosis factor (TNF)α, and interferon (IFN)γ, as well as T cell lymphopenia, decrease of non-classical (CD14^lo^CD16^hi^) monocytes, and occurrence of neutrophil precursors in the peripheral blood of severe COVID-19 patients^3–7^. Older age, male sex, chronic lung and cardiovascular diseases, diabetes mellitus, obesity, host genetics, and IFN autoantibodies have also been associated with severe disease and death^8–11^, but these factors alone do not appear to explain the wide variability in the clinical course of COVID-19.

Mucosal surfaces of the upper respiratory tract and gut are physiologically colonized with a microbiota that consists of trillions of microbial cells and whose diversity and composition vary widely among individuals^12^. The microbiota constantly generates thousands of unique metabolites that can influence many aspects of human biology^13^. Animal studies have revealed that the microbiota calibrates immune responses during pulmonary and systemic infections, e.g., through production of short-chain fatty acids (SCFAs) and tryptophan catabolites, and by deconjugation of primary to secondary bile acids^14–17^. Interindividual gut microbiota differences in humans have been associated with variation in cytokine production capacities of peripheral blood cells^18^, and enrichment of the lung microbiota with oral taxa has been linked to e.g., an enhanced expression of proinflammatory cytokines^19^. Previous studies have also indicated an association between COVID-19 status and/or severity and a reduced gut bacterial diversity with enrichment of opportunistic pathogens, as well as elevated levels of inflammatory cytokines (e.g., IL-1β, IL-6, and CXC chemokine ligand (CXCL)8)^20–22^. Moreover, a reduced abundance of upper respiratory tract commensals in severe COVID-19 patients has been described^23–25^.

To characterize the interplay between the microbiome, metabolome, and immune system during the infection, we collected and deeply phenotyped repeated samples from COVID-19 patients with varying disease severity as well as from uninfected controls. Using a systematic approach to account for clinical and host factors wherever possible, various features of the gut microbiome, immune response, and plasma metabolome were revealed to be robustly associated with SARS-CoV-2 infection, and/or COVID-19 severity.

## Results

**Fig. 1:**
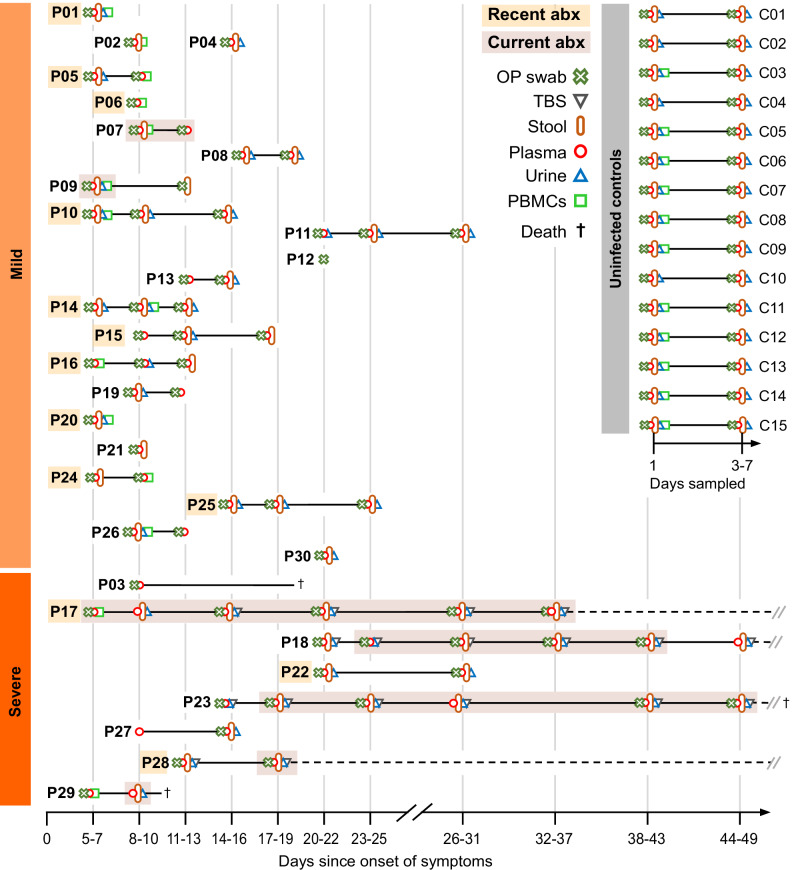
Cohort description and sampling timepoints. Uninfected controls (C1–C15) and enrolled patients (P1–P30) classified by maximum OSCI score. We refer to scores between 1 and 4 as mild and scores between 5 and 8 as severe disease in further discussion. Sampling timepoints are represented according to the days after symptom onset for patients. For uninfected controls, sampling was performed two times, on day 1 and then again 3–7 days after the first sampling. The observation and hospitalization period is marked with a solid line, or a dashed black line when prolonged. Sample materials included oropharyngeal (OP) swabs, plasma, peripheral mononuclear blood cells (PBMCs), urine, stool, and tracheobronchial secretions (TBS). The use of antibiotics shortly before (i.e., recent abx) or during the sampling period (i.e., current abx) is marked for each participant. All control subjects were antibiotic-free for at least 3 months before and during the sampling period.

The present work includes a subset of patients enrolled between March and June 2020 in the Pa-COVID-19 cohort, a prospective observational cohort study of patients with COVID-19 at Charité Universitätsmedizin Berlin^26^. Plasma, stool, urine, and oropharyngeal (OP) swabs from a total of 30 laboratory-confirmed, hospitalized COVID-19 patients with varying degrees of disease severity, as well as 15 uninfected, age- and sex-matched controls were collected (Fig. 1). In parallel, comprehensive clinical information including underlying diseases, medication before and during hospitalization, and the development of secondary infections was obtained (Table 1 and Supplementary Table 1). Patient samples were classified into early or late observation groups based on the number of days since symptom onset (≤10 days or >10 days, respectively). According to the WHO ordinal scale of clinical improvement (OSCI, www.who.int/publications/i/item/covid-19-therapeutic-trial-synopsis), 22 patients (73.3%) had ambulatory to moderate disease (i.e., mild, maximum OSCI score 1–4), and 8 (26.7%) had a severe or critical disease course (i.e., severe, maximum OSCI score 5–8), 3 of whom died in the hospital. The median duration of hospitalization was 8.5 days (range 3–132 days) excluding the patients who died. Peripheral blood mononuclear cells (PBMCs) were obtained from 14 patients at an early phase of infection as well as 11 controls, and tracheobronchial secretions (TBS) were collected from 4 ventilated COVID-19 patients. Whole metagenome sequencing of stool, OP and TBS samples, metabolomics of plasma and urine, single-cell RNA sequencing (scRNAseq) of PBMCs, multiplex cytokine ELISA of plasma, and *IFN* qRT-PCRs of OP samples were performed. Our integrated statistical approach enabled us to analyze -omics and clinical data individually and in conjunction with one another while considering a range of potential confounders.

**Table 1:** Clinical characteristics

Characteristics	Controls	Mild patients	Severe patients
*n*	15	22	8
Average age, y (SD)	52.80 (19.37)	56.09 (18.64)	63.88 (16.47)
Female, % (*n*)	40.0 (6)	45.5 (10)	37.5 (3)
Average BMI, (SD)	23.94 (3.52)	26.8 (5.74)	26.9 (3.53)
Active smokers, % (*n*)	6.7 (1)	4.5 (1)	25.0 (2)
Comorbidities			
Diabetes mellitus, % (*n*)	6.7 (1)	18.2 (4)	25.0 (2)
Cardiovascular disease, % (*n*)	26.7 (4)	50.0 (11)	62.5 (5)
Chronic lung disease, % (*n*)	0 (0)	31.8 (7)	25.0 (2)
Chronic kidney disease, % (*n*)	0 (0)	22.7 (5)	62.5 (5)
Chronic liver disease, % (*n*)	6.7 (1)	9.0 (2)	37.5 (3)
IBD, % (*n*)	0 (0)	4.5 (1)	0 (0)
Dyslipidemia, % (*n*)	13.3 (2)	13.6 (3)	25.0 (2)
Active neoplasia, % (*n*)	0 (0)	0 (0)	12.5 (1)
Altered thyroid hormones, % (*n*)	6.7 (1)	18.2 (4)	37.5 (3)
Charlson Comorbidity Index, median (IQR)	1.0 (3.25)	2.0 (4.0)	3.5 (6.8)
OSCI, median (IQR)	0 (0)	3.5 (1.0)	7.0 (1.0)
Gastrointestinal symptoms at admission, % (*n*)	0 (0)	22.7 (5)	25.0 (2)
Medication, median (IQR)	0 (2.0)	2.0 (3.0)	1.5 (4.75)
Antibiotic therapy			
During sampling period, % (*n*)	0 (0)	18.2 (4)	62.5 (5)
Beta-lactams, %^a^ (*n*)	0 (0)	42.9 (3)	73.3 (11)
Glycopeptides, % (*n*)	0 (0)	0 (0)	20 (3)
Macrolides, % (*n*)	0 (0)	28.6 (2)	0 (0)
Others, % (*n*)	0 (0)	28.6 (2)	6.7 (1)
Evidence of antibiotics at up to 3 months prior, % (*n*)	0 (0)	54.5 (12)	37.5 (3)
Beta-lactams, % (*n*)	0 (0)	26.7 (4)	60.0 (3)
Quinolones, % (*n*)	0 (0)	26.7 (4)	0 (0)
Macrolides, % (*n*)	0 (0)	13.3 (2)	40.0 (2)
Others, % (*n*)	0 (0)	13.3 (2)	0 (0)
Not specified, % (*n*)	0 (0)	20.0 (3)	0 (0)

*SD* standard deviation, *BMI* body mass index, *IQR* interquartile range, *OSCI* ordinal scale for clinical improvement, *IBD* inflammatory bowel disease.

^a^Calculation as % of total of the antibiotics taken (also several per patient).

### Airway and intestinal microbiota disturbance in mild and severe COVID-19

**Fig. 2:**
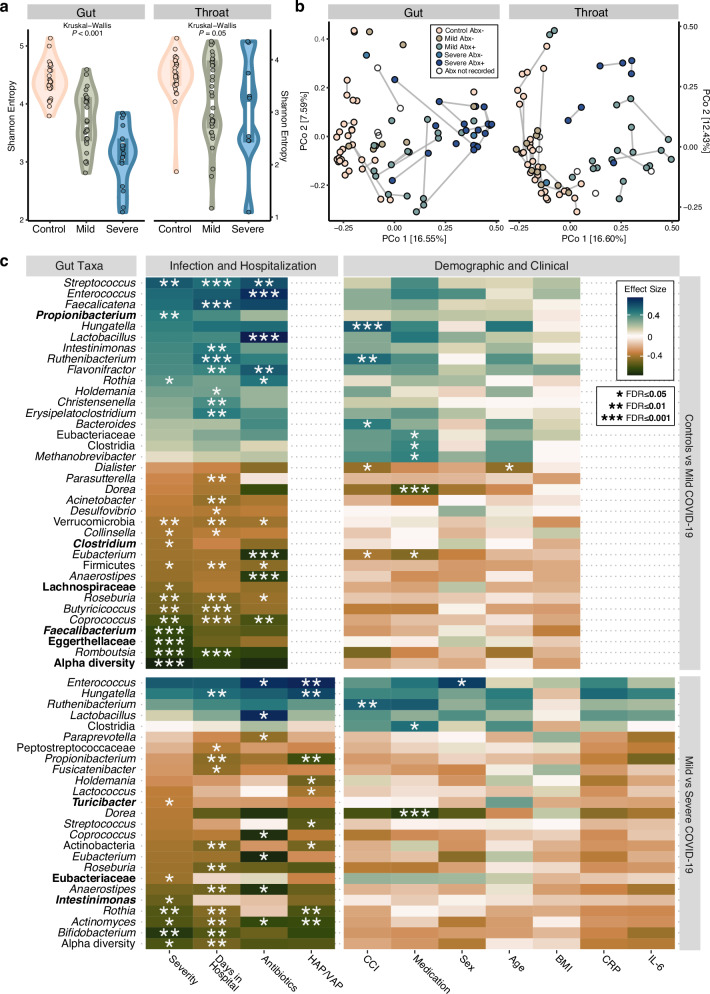
Microbiota compositional changes are associated with COVID-19 severity, hospitalization, and/or antibiotics. **a** Alpha diversity (measured as Shannon entropy) of stool and oropharyngeal samples remaining after rarefaction (see “Methods” and Supplementary Figure 1a, b), separated by disease status and severity (measured by OSCI). Box plots display the median (center line), interquartile range (box bounds), and 1.5 times the interquartile range (whiskers). **b** Beta diversity (principal coordinates analysis, PCoA) on rarefied species abundances, colored to denote disease status and severity as well as any recent or current antibiotic (Abx) intake. **c** Subset of significant results from our differential abundance and confounder testing of the gut microbiota, comparing uninfected controls to mild disease (i.e., status) and mild to severe disease (i.e., severity; see “Methods”, Supplementary Fig. 1c for the throat microbiota, and Supplementary Table 6 for the full results). Standardized, non-parametric effect sizes were calculated between bacterial abundances and clinical covariates (Spearman for continuous or Cliff’s delta/Wilcoxon for binary variables), and tested for significance. Nested linear models and likelihood ratio tests were then used to disentangle the potentially confounding effects of clinical variables from the disease status or severity (on “naively” disease-associated bacterial taxa from the first step), if possible (see “Methods”). Taxa in bold showed a unique association to the group (control, mild or severe COVID-19) which could be disentangled from covariates. “Antibiotics” refers to any recent or current use and “Medication” is a sum of current medications excluding antibiotics. OSCI ordinal scale for clinical improvement, HAP hospital-acquired pneumonia, VAP ventilator-associated pneumonia, CCI Charlson Comorbidity Index, CRP C-reactive protein, IL-6 interleukin 6, FDR false discovery rate (adjusted).

To characterize the microbiota of our cohorts, we conducted shotgun metagenomic sequencing on a total of 94 OP swabs, 18 TBS, and 81 stool samples, as proxies for the throat, pulmonary, and gut microbiota, respectively. After quality control, 75 OP and 72 stool samples remained viable for downstream analysis (see Supplementary Fig. 1a, b). The gut microbiota of COVID-19 patients exhibited significantly decreased alpha diversity compared to uninfected controls (Kruskal-Wallis *P* < 0.001, see Fig. 2a), in line with previous observations^27,28^. Beta diversity analysis appeared to reflect both disease status and severity as well as antibiotic intake (Fig. 2b). Indeed, several bacterial taxa were strongly associated with disease, especially when comparing patients with mild disease to controls, some of which were concurrently associated with the number of days patients were hospitalized (Fig. 2c). Mild courses of COVID-19 most robustly associated with lower abundances of Lachnospiraceae, *Clostridium*, *Faecalibacterium* spp. and Eggerthellaceae in the gut relative to controls, whereas severe cases had decreased *Intestinimonas*, Eubacteriaceae, and *Turicibacter* compared to mild cases (Fig. 2c). Depletion of various other gut commensals such as Firmicutes, *Romboutsia*, *Coprococcus*, and *Roseburia* were additionally associated with longer hospitalization and/or antibiotics. Antibiotic therapy was also associated with an enrichment of *Enterococcus* and *Lactobacillus* in patients with mild or severe COVID-19. Moreover, *Hungatella* abundances were positively correlated with existing comorbidities in mild cases and length of hospital stay and hospital-acquired pneumonia (HAP, referring to all types of nosocomial pneumonia in both ventilated and non-ventilated patients) in severe cases.

In contrast to the intestinal microbiota, compositional differences observed in the oropharyngeal microbiota appeared to be primarily associated with antibiotic intake, rather than disease status or severity (Supplementary Fig. 1c), which can also be observed in the beta diversity analysis (Fig. 2b). Similar to previous findings^29^, the alpha diversity of the oropharyngeal microbiota was lower in COVID-19 patients as compared to uninfected controls, with samples from severe cases showing the most variability among all the groups (Fig. 2a). In addition, a positive correlation between the abundances of Prevotellaceae and *Slackia* and the development of HAP was observed (Supplementary Fig. 1c). Collectively, our results indicate a direct association between intestinal microbiota composition and COVID-19, whereas oropharyngeal microbiota disturbance appeared to be mainly driven by antibiotic use in our patients.

### Immune dysregulation in severe COVID-19

**Fig. 3:**
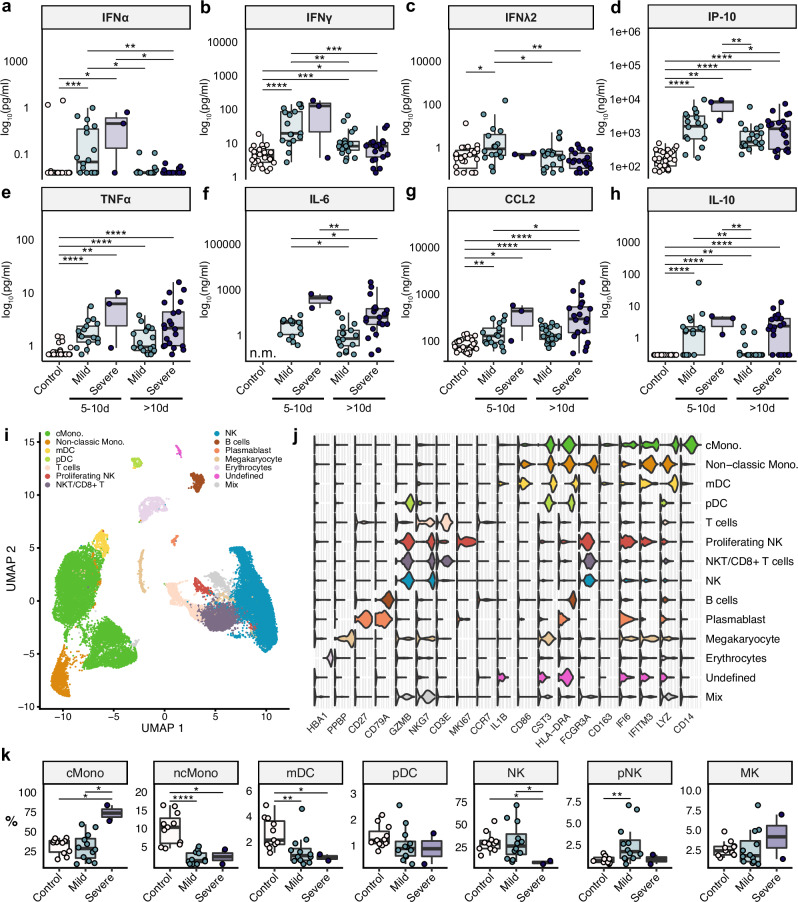
The immune response is dysregulated in severe COVID-19 patients. **a**–**h** Plasma levels of IFNs and inflammatory cytokines in healthy controls, mild and severe COVID-19 patients were measured at two different timepoints after symptom onset (5–10 days and ≥10 days after symptom onset). Box plots display the median (center line), interquartile range (box bounds), and 1.5 times the interquartile range (whiskers). **i** PBMCs from 11 healthy controls and 14 COVID-19 patients (at an early infection phase, i.e., <10 days since symptom onset) were collected, and T and B cells were depleted. UMAP representation of all merged scRNA-seq profiles are shown. 13 cell types were identified by cluster gene signatures. **j** Violin plots showing top marker genes for the cell types shown in (**i**). **k** Relative abundance of major innate immune cells were compared. Their distribution varies between controls and COVID-19 patients and between mild and severe disease. Significant pairwise comparisons are denoted in panels (**a**–**h**) and (**k**) (Mann–Whitney U test). See also Supplementary Figure 2. IFNα interferon alpha, IFNγ interferon gamma, IFNλ2 interferon lambda 2, IP-10 interferon gamma-induced protein 10, TNFα tumor necrosis factor alpha, IL-5 interleukin-5, CCL2 CC-chemokin-ligand-2, IL-10 interleukin-10, n.m. not measured; scRNAseq single-cell RNA sequencing, PBMCs peripheral mononuclear blood cells, cMono classical monocytes, ncMono non-classical monocytes, mDC myeloid dendritic cells, pDC plasmacytoid dendritic cells, NK natural killer cells, NKT natural killer T cells, MK megakaryocytes.

To characterize the systemic immune response in our cohort, we measured cytokines in plasma samples from all patients and uninfected controls. In line with previous studies^29,30^, type I, II and III IFNs as well as several inflammatory cytokines including TNFα, interferon-gamma induced protein (IP)-10/CXCL10, C–C motif chemokine ligand (CCL)2, and IL-10 were increased in early plasma samples of COVID-19 patients when compared to uninfected controls (Fig. 3a–h). While IFN levels mostly decreased in the later phase of the infection, production of the inflammatory cytokines remained high in severe COVID-19 patients. Next, PBMCs from a subset of patients at an early infection time point and uninfected controls were characterized (see Fig. 1) by droplet-based scRNAseq. Since we aimed to focus primarily on the innate immune cells, PBMCs were depleted of T and B lymphocytes before measurements. UMAP and cell type classification identified various cell types and subtypes expected in the mononuclear compartment of blood (Fig. 3i, j). Further analyses revealed an increase of classical monocytes in severe COVID-19 patients as compared to uninfected individuals and patients with mild infection (Fig. 3k), and a depletion of non-classical monocytes and cDCs in early COVID-19. NK cells were only depleted in patients with severe COVID-19. IFN-stimulated genes (ISGs) were highly expressed in PBMCs (Supplementary Fig. 2a, b), their expression positively correlated with systemic levels of both type I and II IFNs (Supplementary Fig. 2c), and they were enhanced in mild and severe COVID-19 patients (Supplementary Fig. 2d). Moreover, expression of type I and III IFN genes in our oropharyngeal samples were measured, and increased *IFNL2* mRNA levels in mild COVID-19 patients as compared to uninfected controls were found (Supplementary Fig. 2e). Overall, our results indicate that the systemic inflammatory response is dysregulated and excessive in patients with severe COVID-19.

### Alterations in tryptophan, bile acid, lipid, and amino acid metabolism in severe COVID-19

**Fig. 4:**
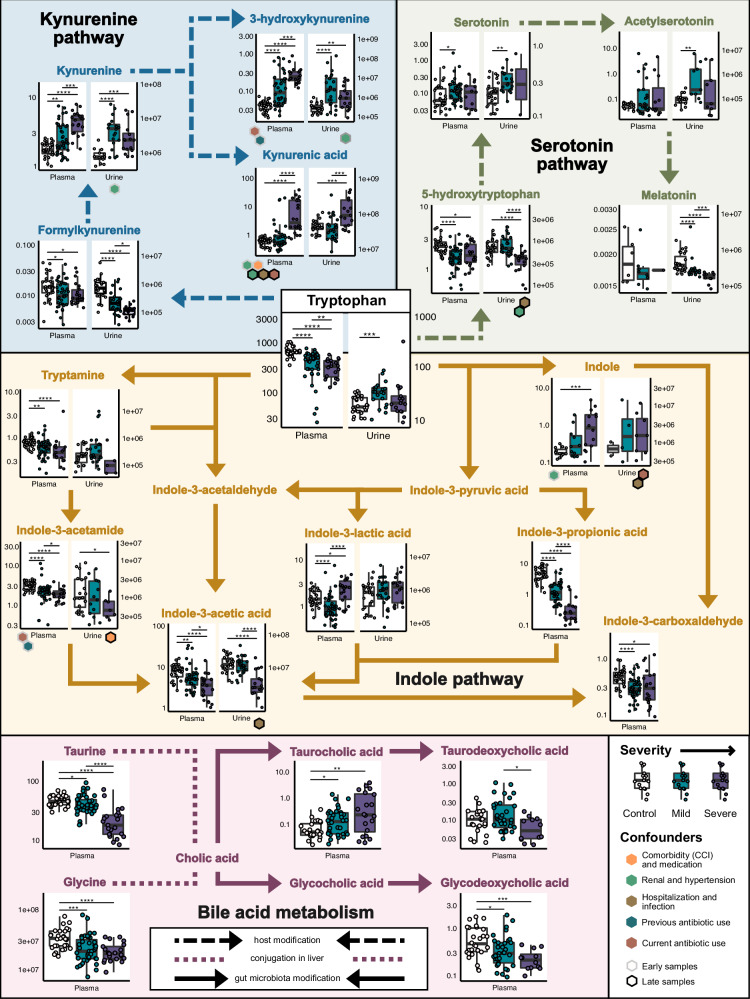
Severe COVID-19 is associated with tryptophan and bile acid metabolites. Tryptophan and bile acid metabolite concentrations (given in ng/mL and µM, respectively) from all plasma and urine samples, annotated with adjusted pairwise Spearman test significance and post hoc identified confounders from early or late slices of the data. Box plots display the median (center line), interquartile range (box bounds), and 1.5 times the interquartile range (whiskers). Some co-associated clinical variables were rationally grouped and relabeled here for annotation purposes, i.e., hospitalization and infection reflects confounding by one or more of the following: HAP, number of days hospitalized, bacteremia and/or sepsis. The kynurenine and serotonin pathways are host-associated, while indole and part of the bile acid metabolism are carried out by gut microbes. See also Supplementary Figure 3. CCI Charlson comorbidity index, HAP hospital-acquired pneumonia.

To identify potential microbiota- and/or host-derived factors underlying COVID-19 phenotypes, we performed metabolomic analyses on a total of 96 plasma and 77 urine samples from COVID-19 patients and uninfected controls. Our analysis highlighted significant differences in the plasma metabolome of COVID-19 patients when compared to uninfected controls, as well as direct associations between various metabolites and disease severity. In COVID-19 patients, lower plasma levels of several tryptophan metabolites including the primarily dietary-derived tryptophan itself (*ρ* = −0.68, FDR-adjusted *P* < 0.0001), the serotonin precursor 5-hydroxytryptophan (*ρ* = −0.38, FDR-adjusted *P* = 0.0003), and the microbial metabolites tryptamine, indole-3-propionic acid, and indole-3-acetic acid were observed^31^ (Fig. 4, Supplementary Fig. 3a), indicating severe disturbance of host-dependent kynurenine and serotonin pathways and the microbiota-dependent indole metabolic pathway. Many of the altered tryptophan metabolites are ligands for the immunoregulatory aryl hydrocarbon receptor (AhR) and/or pregnane X receptor (PXR)^32,33^. In line with previous studies^34,35^, the host-derived tryptophan catabolites kynurenine, which is also an AhR ligand, and the potentially neurotoxic 3-hydroxykynurenine^36^ were strongly enriched in COVID-19 patients, with kynurenine levels showing a robust positive association with severity (*ρ* = 0.7, FDR-adjusted *P* < 0.0001; Fig. 4), in both early and late samples (Supplementary Fig. 3a). Moreover, severe COVID-19 was robustly associated with lower plasma concentrations of the microbiota-produced secondary bile acid glycodeoxycholic acid (*ρ* = −0.42, FDR-adjusted *P* = 0.0006), and with higher levels of the primary bile acid taurocholic acid (Fig. 4). SARS-CoV-2 infection and COVID-19 disease severity were also associated with depletion of various lysophosphatidylcholines at all sampling timepoints and phosphatidylcholines in the early samples (Supplementary Fig. 3a, b). Lysophosphatidylcholines are a group of bioactive lipids with potent proinflammatory and immunoregulatory roles^37^. Taken together, these results demonstrate that tryptophan, bile acid, lipid, and other amino acid metabolism is dysregulated in severe COVID-19.

### Integrated analysis reveals associations between altered levels of tryptophan metabolites and enhanced production of proinflammatory cytokines

**Fig. 5:**
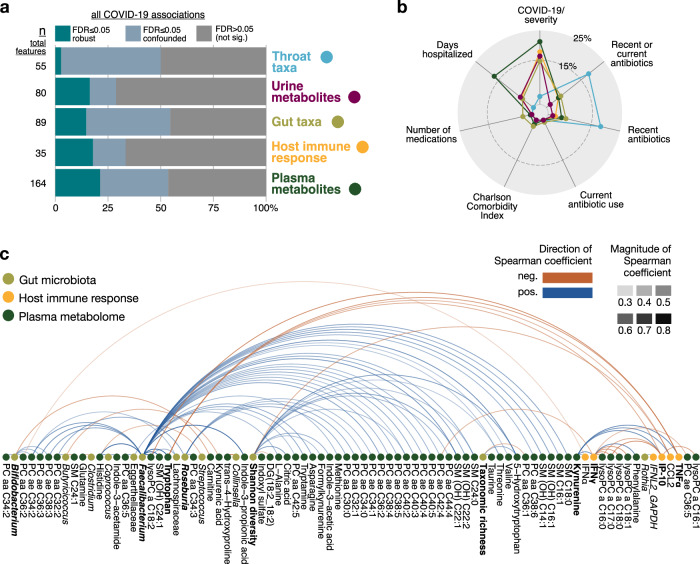
Integration of severity associations across -omics spaces identifies correlated features of the gut microbiome, metabolome, and immune response in (severe) COVID-19. **a** Summary of association classifications with SARS-CoV-2 infection (from comparison between mild COVID-19 and controls) or disease severity (from comparison between mild and severe COVID-19) across all -omics features after confounder analysis. Plasma metabolites had the highest percentage of total features which were robustly associated with SARS-CoV-2 infection and/or COVID-19 severity. **b** Main confounding clinical variables for all significant disease or severity associations are shown (i.e., cumulative area of non-gray bars from (**a**)), as well as an estimate of the percentage of those which were confounded, and if so by what. **c** Robust associations (FDR ≤ 0.05) between the gut microbiome, plasma metabolome, and host immune response from the subset of features associated with SARS-CoV-2 infection or COVID-19 severity in (**a**). Bolded features had more than three robust associations with features from another -omics space.

Finally, we integrated our various -omics data into our mixed-models analysis framework in order to establish associations between the microbiome, metabolome, and immune response parameters. First, we summarized the extent to which features from the different -omics spaces were robustly associated with SARS-CoV-2 infection and/or disease severity, or confounded by different clinical factors such as previous or current antibiotic use, other medications, comorbidities, and days of hospitalization. This analysis revealed that several features of the gut microbiome, immune response, and metabolome were robustly associated with COVID-19 severity, whereas almost all features of the oropharyngeal microbiome were only indirectly (i.e., confounded) or not significantly associated with COVID-19 severity (Fig. 5a, b). Many associations of the oropharyngeal microbiome were rendered non-significant (i.e., were confounded) by recent antibiotic therapies (Fig. 5b). There were hardly any associations with demographic factors such as age, sex, or BMI (also demonstrated in Fig. 2c, Supplementary Fig. 1c). To identify associations between features from different -omics spaces, the robust infection- and severity-associated subsets from the gut microbiome, plasma metabolome, and host immune response were then used in further modeling steps (see “Methods”). This analysis uncovered 84 associations between the gut microbiome and the plasma metabolome, 2 between the gut microbiome and the immune response, and 30 between the plasma metabolome and the immune response (Fig. 5c). Notably, *Faecalibacterium* was strongly positively correlated with tryptophan and several of its metabolites including 5-hydroxytryptophan, tryptamine, and indole-3-propionic acid, many of which have immunomodulatory activities through their effects on AhR and/or PXR^32,33^. Depletion of *Faecalibacterium* might thus be at least partly responsible for the reduced levels of these tryptophan metabolites. At the same time, this taxon was strongly positively correlated with several phosphatidylcholines and other plasma lipids, as well as histidine and threonine. Plasma kynurenine was strongly positively correlated with several proinflammatory cytokines, whereas tryptophan was negatively correlated with those same cytokines, including IFNγ, TNFα, IP-10, and/or CCL2. *Bifidobacterium* was positively correlated with several phosphatidylcholines and negatively correlated with carnitine. Lower alpha diversity (measured by the Shannon entropy) was associated with lower levels of plasma 5-hydroxytryptophan and formylkynurenine and higher levels of CCL2. Moreover, IFNγ production was positively correlated with phenylalanine. Taken together, our analysis indicates that alterations in both the microbiota- and host-dependent tryptophan metabolism, as well as potentially other metabolic pathways, may contribute to the dysregulated inflammatory immune reaction in severe COVID-19.

## Discussion

While several excellent microbiomics^20,24,27,38,39^, metabolomics^34^, and multi-omics studies^35,40–46^ of COVID-19 have been published, our work is unique in simultaneously measuring and analyzing a particularly large number of different -omics features, and, in this, to integrate gut and oropharyngeal metagenome sequencing, metabolomics, host transcriptomics, and cytokine profiling. Our analyses using linear mixed-effect models and exhaustive confounder testing revealed the plasma metabolome to be the -omics domain most affected by SARS-CoV-2 infection. Consistent with previous observations^34,40,47^, plasma levels of various host- and microbiota-derived tryptophan metabolites and lysophosphatidylcholines robustly correlated with COVID-19 severity, as did secondary bile acids in our study. In addition, enhanced inflammatory cytokine production and gut microbiota perturbations were strongly associated with the infection. For example, taxonomic diversity in the gut was diminished in COVID-19 patients, and several potentially beneficial commensals (mainly belonging to the Clostridiales order) were depleted, which is consistent with previous reports^20,21^. While depletion of some gut commensals (e.g., Lachnospiraceae, *Clostridium* spp., *Faecalibacterium* spp., Eggerthellaceae, *Intestinimonas*, Eubacteriaceae, and *Turicibacter*) was directly associated with disease status or severity, changes in the abundance of others (e.g., *Romboutsia*, *Coprococcus*., *Bifidobacterium* spp.) were additionally linked to length of hospitalization and/or intake of antibiotics.

In addition to identifying potential microbial and clinical biomarkers of SARS-CoV-2 infection, we propose mechanistic hypotheses into the dysregulated immune response considered causative of severe COVID-19. For example, we related the depletion of *Faecalibacterium* to decreased levels of various tryptophan metabolites, many of which are known as immunoregulatory and as ligands of AhR and/or PXR^32^. The association between *Faecalibacterium* and tryptophan metabolisms has been described before in the context of other conditions^48,49^. Moreover, decrease in tryptophan and increase in kynurenine levels, which is commonly linked to the activity of the enzyme IDO1^33^, were associated with enhanced production of several proinflammatory cytokines. The correlation between decreased tryptophan, increased kynurenine levels and higher concentrations of proinflammatory cytokines such as IFNγ in COVID-19 patients has also been described in other studies^34,50–52^. In hospitalized patients, we estimated these mechanisms to further involve a vicious cycle, as critical illness, prolonged hospitalization, and high concentrations of inflammatory mediators further exacerbate the disruption of the microbiome and metabolome. Still, we speculate that several of our findings, e.g., hypotheses describing how certain intestinal commensals are associated with specific metabolites, or how tryptophan catabolites may regulate systemic cytokine production, are also relevant for other types of severe infections and perhaps non-infectious inflammatory diseases.

In accordance with previous studies^34,35^, levels of the host-derived tryptophan catabolite kynurenine were strongly elevated in severe COVID-19 patients. In contrast, tryptamine, indole-3-acetic acid, and other microbiota-derived tryptophan catabolites were depleted in these patients. All of these metabolites are known to activate AhR and/or PXR, which controls the differentiation and inflammatory potential of various innate and adaptive immune cells^31,33,53^. It is likely that, in aggregate, the markedly altered levels of these AhR and PXR ligands observed in our cohort contributed to the dysregulation of the immune response in severe COVID-19; however, further studies are needed to understand the cumulative impact of oppositely altered tryptophan catabolites (which presumably also differ with respect to their AhR and PXR binding affinities) on individual immune cells. Moreover, more research is also required to characterize the impact of these metabolites in different phases of COVID-19, such as the acute inflammatory phase, resolution, or the subsequent period of tissue repair.

From our integrated statistical modeling, it is possible to consider that the depletion of 5-hydroxytryptophan was potentially mediated by low intestinal abundances of *Faecalibacterium* spp., which is consistent with previous findings about the role of the microbiota in controlling the production of 5-hydroxytryptophan by colonic enterochromaffin cells^54^. 5-hydroxytryptophan has also recently been described to activate AhR and to mediate CD8+ T cell exhaustion in antitumor immunity^55^. Thus, it appears reasonable to speculate that 5-hydroxytryptophan contributes to AhR-mediated calibration of inflammatory cytokine production during COVID-19, as well as to T cell exhaustion characteristic of severe SARS-CoV2 infection^56,57^. Moreover, reduced levels of indole-3-propionic acid, previously shown to be produced by *Clostridium sporogenes*^58,59^, correlated with *Faecalibacterium* spp. depletion. Interestingly, indole-3-propionic has recently been implicated in protection against influenza infection in mice^60^.

We observed reduced levels of lysophosphatidylcholines in severe COVID-19 patients, which is in line with previous studies^40,47,61^, and strong associations with enhanced IFNγ. Lysophosphatidylcholines are a group of bioactive lipids that are produced from phosphatidylcholine by the enzyme phospholipase A2, and shown to have effects on e.g., endothelial cells and immune cells^62^. Low plasma levels of lysophosphatidylcholine have been associated with unfavorable outcomes in several chronic diseases^37^ and sepsis^63^. Moreover, lysophosphatidylcholine treatment was protective in mouse models of sepsis^64^.

Another interesting group of metabolites whose production we found to decrease with severe COVID-19 was the secondary bile acids. Secondary bile acids, which are converted from liver-derived primary bile acids by the microbiota, are known for their ability to influence various immune cells, e.g., via the receptors TGR5 and FXR^65–68^. Indeed, a previous study uncovered how secondary acids control immunity against Chikungunya virus by enhancing type I IFN production by pDCs^65^. Moreover, decreased concentrations of secondary bile acids in fecal samples of patients with severe COVID-19 have previously been associated with mortality^69^. Some studies also indicated that treatment with secondary bile acids could be protective in COVID-19, whereas others did not observe such beneficial effect^70–73^. Further studies are required to evaluate the impact of secondary bile acids and other microbiota-derived metabolites on the immune response during COVID-19.

Our multi-omics study explored alterations in the microbiome, metabolome, and immune response observed in severe COVID-19, and generated several testable hypotheses, but is not without limitations. First, only a relatively small number of patients from a single center were included, which mandates future validation in larger cohorts of patients. Second, for practical reasons and similar to probably all previously published work, we were unable to collect samples during the first days of infection, making it impossible to draw conclusions about mechanisms in the early phase of COVID-19. Moreover, we did not have information regarding dietary habits of the patients as well as nutritional information from their time in the hospital, both of which have been shown to play a role in the gut microbiota composition. Fourth, we lacked an intensive care control group that may have enabled us to better disentangle critical disease and hospitalization-associated factors from COVID-19 specifically. Finally, analyses of the immune response in the lung as the epicenter of the infectious event in COVID-19 were not performed, which would be important for future studies, and many of our respiratory (TBS and some OP) microbiota samples contained too few reads for downstream analysis (Supplementary Fig. S1). Future work pursuing more translational aims (e.g., to predict or possibly treat severe COVID-19) would need to address these limitations, for example via animal experiments to mechanistically explore the microbiome-metabolite-immune networks in COVID-19, and perhaps other infectious and inflammatory diseases. Despite the limitations regarding our study design, we were able to perform deep phenotyping using various state-of-the-art -omics techniques and clinical metadata, allowing us insight into interactions between the microbiome, metabolome, and immune system network in general, and into the pathogenesis of severe COVID-19 in particular. The disrupted microbiome-tryptophan metabolism-immune network described here might represent a potential target for intervention strategies to protect patients from severe COVID-19.

## Methods

### Study design and patient inclusion criteria

In the framework of the Pa-COVID-19, a prospective observational cohort study of patients with confirmed SARS-CoV-2 infection treated at Charité-Universitätsmedizin Berlin, we collected repeated stool, urine, TBS, and blood samples as well as oropharyngeal swabs from hospitalized patients with COVID-19^26^. All patients with SARS-CoV-2 infection, as determined by positive PCR from respiratory specimens, who were hospitalized at the Charité-Universitätsmedizin Berlin between March and June 2020 and were willing to provide written informed consent were eligible for inclusion. Exclusion criteria included refusal to participate in the clinical study by patient or legal representative or clinical conditions that did not allow for blood sampling. The patients included in this study were enrolled between March 21 and June 15, 2020, before vaccinations or variants of concern. COVID-19 disease severity was classified to mild or severe disease according to the WHO clinical ordinal scale (https://www.who.int/publications/i/item/clinical-management-of-covid-19). The Pa-COVID-19 and COV-IMMUN studies are carried out according to the Declaration of Helsinki and were approved by the ethics committee of Charité-Universitätsmedizin Berlin (EA2/066/20, EA1/068/20). All patients or their legal representatives as well as the healthy individuals provided written informed consent for participation in the study.

### PBMCs isolation and scRNA sequencing

PBMCs were isolated from heparinized whole blood by density centrifugation over Pancoll and cryopreserved in liquid nitrogen until further analysis. Frozen PBMC were recovered by rapidly thawing, and T and B cells were depleted by using CD19 and CD3 MicroBeads (Miltenyi Biotec Cat#130-097-055 and #130-097-043) to enrich for myeloid cells. Subsequently, the PBMC samples were hash-tagged with TotalSeq-A^TM^ antibodies (Biolegend) and scRNAseq was performed by using a droplet-based single-cell platform (10xGenomics) as described recently^4^.

### ScRNAseq data analysis

The 10x Genomics CellRanger pipeline (v4.0.0) was used to pre-process the sequencing data. In brief, BCL files from each library were converted to FASTQ reads using bcl2fastq Conversion Software (Illumina) using the respective sample sheet with the 10x barcodes and TotalSeq antibodies utilized. Then, the reads were further aligned to the reference genome provided by 10× Genomics (Human reference dataset refdata-cellranger-GRCh38-3.0.0) and a digital gene expression matrix was generated to record the number of UMIs for each gene in each cell. Next, the expression matrix from each library was loaded into R/Seurat packages^74^ (v4.0.1) for downstream analysis. To control the data quality, we further excluded low-quality cells with >15% mitochondrial reads, <100 or >3000 expressed genes, or <500 UMI counts. In addition, genes expressed in less than three cells were also excluded from further analysis. After QC, we normalized the gene counts from each cell, where original gene counts were divided by total UMI counts, multiplied by 10,000 (TP10K), and then log-transformed by log10(TP10k + 1). We then scaled the data, regressing for total UMI counts, and performed principal component analysis (PCA) based on the 2000 most-variable features identified using the vst method implemented in Seurat. Cells were then clustered using the Louvain algorithm based on the first 20 PC dimensions with a resolution of 0.3. For visualization, we applied UMAP based on the first 20 PC dimensions. The obtained clusters were annotated by the expression of PBMC marker genes. The expression of selected genes was visualized by violin plots.

### Quantitative reverse transcription PCR

For measuring expression of type I and II IFNs in the upper airways, total RNA was isolated from oropharyngeal swab fluid using mirVana™ miRNA Isolation Kit (Cat# AM1561). The RNA was reverse-transcribed using the high capacity reverse transcription kit (Applied Biosystems, Darmstadt, Germany), and quantitative PCR was performed using TaqMan assays (*GAPDH*: Hs02786624_g1, *IFNL2*: Hs04193048_gH, *IFNB*: Hs01077958_s1 Life Technologies, Darmstadt, Germany) on an ABI 7300 instrument (Applied Biosystems, Darmstadt, Germany). The input was normalized to the average expression of GAPDH and relative expression (relative quantity, RQ) of the respective gene in the healthy control individuals was set as 1.

### Cytokine ELISA

Plasma concentrations of IL-10, IL-12p70, IL-17A, IL-1α, IL-1β, IL-4, IL-22, IP-10, MCP-1, TNFα, and IFNγ were measured by using MSD Meso Scale V-Plex assay kits (Meso Scale Diagnostics). Plasma concentrations of IFNα and IL-28A were quantified by using Simoa® Technology (Quanterix Corporation). Samples were diluted 1:2 (IFNγ, IL-10, IL12p70, IL-1α, IL-1β, TNFα, IL-4) or 1:4 (IP-10, MCP-1, IL-17, IL-22) prior to analysis and processed according to the manufacturer’s instructions.

### Viral load measurements

SARS-CoV-2 RNA detection and quantification in respiratory swabs and stool samples was done as described before^75,76^ and by using either the cobas® SARS-CoV-2 test on the cobas® 6800/8800 system or the SARS-CoV-2 E-gene assay from TibMolbiol on a Roche MagNApure 96/LightCycler 480er workflow. Viral RNA concentrations were calculated by using the CT-Value of the E-gen target, oligo binding site within the E gene and corresponding PCR fragment was described in Corman et al.^76^ and by applying calibration curves of quantified reference samples and in vitro transcribed RNA^77,78^.

### Plasma and urine metabolomic sample pre-processing

Plasma and urine samples were prepared in four different ways depending on the metabolite of interest. Urine samples were treated with urease before Biocrates and CCM analyses. A broad metabolite analysis was conducted using a Biocrates MxP Quant 500 kit. For safety reasons, samples were measured after adding 100% ethanol (LC-MS grade; Fisher Scientific) to the plasma and urine samples. For the analysis of tryptophan derivatives, an extraction solvent (89.9% Methanol in 0.2% FA and 0.02% ascorbic acid) was added to the plasma and urine samples. The preparation of the plasma and urine samples for CCM GC-MS analysis consisted of adding 100% Methanol (LC-MS grade; Fisher Scientific).

### Biocrates MxP Quant 500 assay and measurement

Plasma or urine was added in a 1:2 dilution to ethanol (EtOH, Fisher Scientific, New Hampshire, USA; 50 µL to 100 µL EtOH) and vortexed for 20 s. Samples were stored at −80 °C until use. The MxP Quant 500 kit from Biocrates Life Science AG is a fully automated assay based on phenylisothiocyanate (PITC) derivatization of the target analytes using internal standards for quantitation. Plate preparation was done according to the manufacturer’s protocol. Briefly, 30 µL of the diluted plasma or urine was transferred to the upper 96-well plate and dried under a nitrogen stream. Thereafter, 50 µL of a 5% PITC solution was added. After incubation, the filter spots were dried again before the metabolites were extracted using 5 mM ammonium acetate in methanol (MeOH, Fisher Scientific, New Hampshire, US) into the lower 96-well plate for analysis after further dilution using the MS running solvent A. Quality control (QC) samples were prepared by pooling plasma or urine from each sample.

Evaluation of the instrument performance prior to sample analysis was assessed by the system suitability test (SST) according to the manufacturer’s protocol. The LC-MS system consisted of a 1290 Infinity UHPLC-system (Agilent, Santa Clara, CA, USA) coupled to a QTrap 5500 (AB Sciex Germany GmbH, Darmstadt, Germany) with a TurboV source. Quality assurance and control were reported using the recommended standards by mQACC (Supplementary Table 8). Acquisition method parameters and UHPLC gradient for LC and FIA mode are shown in Supplementary Tables 9–11. All compounds were identified and quantified using isotopically-labeled internal standards and multiple reaction monitoring (MRM) for LC and full MS for FIA as optimized and raw data was computed in Met*IDQ*^TM^ version Oxygen (Biocrates Life Science AG, Innsbruck, Austria). A script developed in-house (MetaQUAC) was used for data quality analysis and preprocessing^79^.

### Gas chromatography mass spectrometry (GC-MS) measurement of key central carbon pathway metabolites

MeOH containing 2 µg/mL cinnamic acid as internal standard (Sigma Aldrich, St. Louis, Missouri, USA) was aliquoted (112.5 µL) and stored on ice. 25 µL of plasma was added to the MeOH followed by addition of 329 µL MeOH, 658 µL chloroform (CHCl_3_, Sigma Aldrich, St. Louis, Missouri, USA), and 382.5 µL water (H_2_O, Fisher Scientific, New Hampshire, USA). Samples were vortexed and left on ice for 10 min to separate into a biphasic mixture. The samples were centrifuged at 2560 × *g* for 20 min at 4 °C and then left to equilibrate at room temperature for 20 min. 300 µL of the upper polar phase was then collected and dried in a rotational vacuum concentrator (Martin Christ, Osterode, Germany). To the urine samples (150 µL), 200 µL of 1 mg/mL urease solution in water was added, sonificated for 15 min and left on ice for 45 min. Ice cold MeOH (800 µL containing 2 µg/mL cinnamic acid as internal standard) was added, vortexed and centrifuged at maximum speed for 10 min at 4 °C. The supernatant (750 µL) was transferred to a new vial and stored at −80 °C until use. Urine samples were normalized to the according osmolarity and dried in a rotational vacuum concentrator (Martin Christ, Osterode, Germany). Quality control (QC) samples were prepared by pooling the extracts of plasma or urine from each sample.

For derivatization the extracts were removed from the freezer and dried in a rotational vacuum concentrator (Martin Christ, Osterode, Germany) for 60 min before further processing to ensure there was no residual water which may influence the derivatization efficiency. The dried extracts were dissolved in 15 µL or 20 µL of methoxyamine hydrochloride solution (40 mg/mL in pyridine, both Sigma Aldrich, St. Louis, Missouri, U) and incubated for 90 min at 30 °C with constant shaking, followed by the addition of 50 µL or 80 µL of N-methyl-N-[trimethylsilyl]trifluoroacetamide (MSTFA, Macherey-Nagel, Düren, Germany) and incubated at 37 °C for 60 min for plasma and urine, respectively. The extracts were centrifuged for 10 min at 18,213 × *g*, and aliquots of 25 µL (plasma) or 30 µL (urine) were transferred into glass vials for GC-MS measurements. QC samples were prepared in the same way. An identification mixture for reliable compound identification was prepared and derivatized in the same way, and an alkane mixture for a reliable retention index calculation was included (10.3390/metabo10110457). The metabolite analysis was performed on a Pegasus 4D GCxGC TOFMS-System (LECO Corporation) complemented with an auto-sampler (Gerstel MPS DualHead with CAS4 injector). The samples were injected in split mode (split 1:5, injection volume 1 µL) in a temperature-controlled injector with a baffled glass liner (Gerstel). The following temperature program was applied during the sample injection: for 2 min, the column was allowed to equilibrate at 68 °C, then the temperature was increased by 5 °C/min until 120 °C, then by 7 °C/min up to 200 °C, then by 12 °C/min up to a maximum temperature of 320 °C, which was then held for 7.5 min. The gas chromatographic separation was performed on an Agilent 7890 (Agilent Technologies), equipped with a VF-5 ms column (Agilent Technologies) of 30 m length, 250 µm inner diameter, and 0.25 µm film thickness. Helium was used as the carrier gas with a flow rate of 1.2 mL/min. The spectra were recorded in a mass range of 60 to 600 *m*/*z* with 10 spectra/second. Each sample was measured twice (technical replicates). The GC-MS chromatograms were processed with the ChromaTOF software (LECO Corporation) including baseline assessment, peak picking, and computation of the area and height of peaks without a calibration by using an in-house created reference and library containing the top 3 masses by intensity for 42 metabolites (55 intermediates; Supplementary Table 12) related to the central carbon metabolism.

The data were exported and merged using an in-house written R script. The peak area of each metabolite was calculated by normalization to the internal standard cinnamic acid. Relative quantities were used. CCM and tryptophan data were batch corrected using the cubic^80^ spline drift correction from notame (v0.0.5, in R v4.0.1) followed by QC-sample median normalization. Urine tryptophan data was only QC-sample median normalized. Quality assurance and control were reported using the recommended standards by mQACC (Supplementary Table 8).

### Tryptophan metabolite analysis using UPLC-MS

For the tryptophan analysis, liquid chromatography – mass spectrometry (LC-MS) analysis was performed with a 1290 Infinity 2D HPLC system (Agilent Technologies, USA) combined with a TSQ Quantiva triple quadrupole mass spectrometer with a heated ESI source (Thermo Scientific, USA). Before starting, an extracting solvent was prepared comprising 90% methanol, 0.15 µg/mL mixed internal standards, 0.02% ascorbic acid, and 0.2% formic acid. This was placed at −20 °C to cool. For urine samples a 1:5 (v/v) dilution was prepared in water prior to a urease digestion at 37 °C for 40 min with 10U urease (Sigma Aldrich). For each sample, 280 µL pre-chilled extracting solvent was added to 140 µl of plasma or urease digested urine. Samples were held at 4 °C and shaken for 10 min at 1000 rpm (Eppendorf ThermoMixer C), incubated at −20 °C before being centrifuged for 15 min at 11,000 × *g* and 4 °C. The supernatant was transferred to a dark LC-MS vial for LC-MS/MS analysis. 20 µl of each plasma sample was pooled, and the pooled plasma was also extracted to make quality control (QC) samples. These QC samples were run every 6 samples. LC-MS analysis of 5 µl injection was combined with a triple quadrupole mass spectrometer using a 10-min gradient. A reversed-phase column was used (VisionHT C18 Basic; L × I.D. 150 mm × 4.6 mm, 3 μm particle size, Dr Maisch, Germany) and held at a constant temperature of 30 °C. The mobile phase consisted of 0.2% formic acid in H_2_O (solvent A) and 0.2% formic acid in methanol (solvent B). The following gradient was run with a constant flow rate of 0.4 ml min^−1^: A/B 97/3 (0 min), 70/30 (from 1.2 min), 40/60 (from 2.7 to 3.75 min), 5/95 (from 4.5 to 6.6 min), and 97/3 (from 6.75 to 10 min). The molecular ion and at least two transitions were monitored for the 15 metabolites that are part of the tryptophan pathway.

Data was exported into Skyline (v.19.1, 64-bit) to identify and quantify peak intensity and area. Transition settings in the Skyline search were: isotopic peaks included: count; precursor mass analyzer: QIT; acquisition method: targeted; product mass analyzer: QIT. Method match searching tolerance was 0.6 *m*/*z*, and data was manually checked to ensure the correct peaks were selected. Cubic spline drift correction was applied per metabolite and to all sample types using the pooled quality control (QC) samples as references to fit the splines. The first and the last QC samples used to fit the cubic splines are the most critical to the resulting fit. In this case, the last conditioning pooled QC sample and the first of two pooled QC replicates at the end of the analytical run were used as the first and last QC respectively in the batch correction. Standard samples of increasing concentration were used to construct calibration curves using linear fits per metabolite in ng/ml. Concentration values less or equal to zero were declared as missing. In this study, several study groups featured measurements systematically outside of the calibration range for some metabolites (i.e., below or above the smallest or largest standard sample applied for a metabolites calibration curve, respectively). Further, calibration of QC standard samples resulted in insufficient accuracy. However, precision (%RSD in either standard or pooled QC samples) was adequate for most compounds. Hence, metabolites cannot be considered as absolutely but as relatively quantified in this study. In-house R scripts were used for internal standard normalization, calibration, statistics, and plotting. Quality assurance and control were reported using the recommended standards by mQACC (Supplementary Table 8).

### Metagenomic sample pre-processing, DNA extraction, and sequencing

Oropharyngeal swabs and stool samples were collected in collection tubes containing DNA/RNA shield (Zymo Research Cat# R1107-E and Cat# R1101) and frozen at −80 °C until further analysis was performed. DNA was isolated from the oropharyngeal swabs and stool samples using the ZymoBIOMICS™ DNA Miniprep Kit (Cat#D4300). For DNA isolation lysis of microbes was performed by mechanical disruption using a Mini-BeadBeater-96 (BioSpec) two times for 2 min. Libraries were prepared using 250 ng DNA as input for the NEBNext Ultra II DNA Library Prep Kit (NEB Biolabs) according to manufacturer’s instructions. Sequencing was performed on the Illumina NovaSeq platform (PE150) at an average sequencing depth of 5.6 Gbp.

### Taxonomic microbiome profiling

Whole genome shotgun sequencing reads were analyzed using the NGLess pipeline (v1.3.0). Sequences were quality controlled, trimmed (Phred <25), filtered (length <45 bp) and merged using NGLess defaults and subsequently filtered for human reads (reference GRCh38; low complexity regions and regions mapping to the progenomes 2 gene catalog were masked using bbtools; minimum match size = 45 bp, minimum identity = 90%). One stool sample and two TBS samples were of insufficient quality and removed from further analysis. Taxonomic assignment was performed using the mOTUs profiler (v2.6).

### Taxonomic profile pre-processing, normalization, and diversity analyses

For alpha and beta diversity analysis, stool and oropharyngeal sample mOTUs were first rarefied to correct for sequencing depth variation (10 K reads/sample for stool and 600 reads/sample for oropharyngeal samples), resulting in 75 and 72 samples, respectively. Sequencing depths were too low to proceed for TBS samples (see Supplementary Fig. 1). The *vegan* (v2.5.7) and *stats* packages were used for alpha and beta diversity calculations on these mOTU counts. For differential abundance and confounder testing with linear models, as stated where results are referenced, manually-binned genus-level mOTU counts were used to increase the strength of the signal given the small sample size, which the integration analysis included an additional filtering step to further refine (more detail in that section, below). Rarefied counts were binned, transformed to relative abundances, then filtered to exclude features which were (a) nonzero in less than 20% of samples, (b) with a mean relative abundance less than 10e−4, or (c) with zero variance, and finally (d) log-transformed before linear modeling^81^.

### Statistical testing of -omics data and post hoc confounder analysis with clinical variables

All statistical analysis was performed with R (v4.0.3) using the *targets* workflow manager (v0.12.1) and *renv* environment manager (v0.12.5) to enhance reproducibility. All figures were generated using *ggplot2* (v3.3.3) and *patchwork* (v1.1.1). Testing was performed using the *metadeconfoundR* package (v0.2.7) as described in Forslund et al.^82^ (especially Extended Data Fig. 1 for a graphical overview) and briefly described here.

To first identify which clinical variables associated with -omics features, standardized, non-parametric effect sizes (Cliff’s delta and the Spearman correlation for binary and continuous variables, respectively) were calculated and tested for significance. The full set of clinical variables and per-individual values are given in Supplementary Table 1; variables which had less than three nonzero observations in both groups being compared (i.e., 6 total between uninfected controls and mild COVID-19, or mild and severe COVID-19) were not tested.

In a second step, significant clinical variables from the first step were used in an iterative, nested regression procedure to assess post-hoc confounding potential. Single -omics feature abundances were rank-transformed and regressed onto a binary disease status label, both (1) with and (2) without a potentially confounding variable identified from the first step, followed by a likelihood ratio test (LRT) between nested models 1 and 2. Linear mixed-effect models were used to account for repeated sampling (i.e., the formula included a + (1|PatientID) term). This was repeated combinatorially across all post-processed -omics feature abundances and clinical variables, and integrated to yield a single status for each feature-clinical variable association (including disease status and severity): robust (not confounded by any naively significant covariates), confounded (and if so by what/which), or not significant (summary of results shown in Fig. 5a). The Benjamini-Hochberg procedure was used to correct for multiple testing in both the naive statistical tests and likelihood ratio tests.

Concretely, our software produces a table of results in which each row contains the statistical summary for a single -omics feature and clinical variable pair (e.g., alpha diversity and OSCI score, respectively). There are identifier columns for each of these (“Y_dep.var” and “X_ind.var” for dependent Y and independent X variables in the models, respectively), and columns for naive effect size and adjusted *p*-values (XY_eff.size and XY_p.adj, respectively). The final column (“AssocStatus”) is either a status (D for “deconfounded” or NS for “not significant”), or, if “confounded”, a list of other clinical variables which resulted in a no-longer-significant association between X and Y in the given row when modeled as second independent variable. In our example, the gut alpha diversity was “deconfounded” (i.e., robustly associated with the OSCI score), while the oropharyngeal alpha diversity was confounded by antibiotic use, which was listed (see Supplementary Fig. 1). Our combined results from analysis with each of the post-processed -omics data tables and clinical metadata are given in Supplementary Table 6.

### Cross-omics associations and integrated statistical analysis

As described above, all individual -omics features were tested for associations with the same set of clinical factors including disease status (in the case of uninfected controls vs mild COVID-19) and severity (in the case of mild vs severe COVID-19), revealing a subset of features from each space which was robustly correlated with the OSCI score (Fig. 5a, b). To examine associations and generate hypotheses between different -omics spaces, we reconfigured our statistical framework to include robust subsets of severity-associated “cross-omics” features as additional independent variables, analogous to the way clinical variables were previously treated. This produced naive correlations between e.g., disease-associated microbial taxa and metabolites or immune parameters, and further expanded our ability to classify their robustness via iterative nested model testing.

As a concrete example of a single step in this extended framework: plasma kynurenine and IFNγ were both robustly associated with severity, so kynurenine was included as an additional independent variable when re-testing IFNγ against disease status or severity. Three models were built:

osci_model: rank(IFNγ) ~ disease_status

kyn_model: rank(IFNγ) ~ kynurenine

full_model: rank(IFNγ) ~ disease_status + kynurenine

Then two likelihood ratio tests (LRTs) were performed with different nested model comparisons, and their results were used to classify the association between IFNγ and the disease status:

Test 1: likelihood ratio test between full_model and osci_model

Test 2: likelihood ratio test between full_model and kyn_model

Test 1 checks whether kynurenine explains significant variation in IFNγ measurements beyond that which is already explained by the presence or severity of disease (again depending on the comparison being carried out), while test 2 checks the converse. If only test 1 is significant, then, it can be concluded that the disease-IFNγ association is statistically reducible to the kynurenine-IFNγ association, and therefore the disease-IFNγ association may be considered “confounded” by kynurenine. If only test 2 or both tests are significant, the disease-IFNγ association is at least partially statistically independent of kynurenine, and may be considered robust (so long as it remains statistically independent from the other clinical and cross-omics variables tested). Our combined results needed to generate Fig. 5c are given in Supplementary Table 7.

### Reporting summary

Further information on research design is available in the Nature Research Reporting Summary linked to this article.

### Supplementary information

supplementary information

Reporting summary

## Data Availability

Raw sequencing data have been deposited under BioProject accession number PRJNA909223 (www.ncbi.nlm.nih.gov/bioproject/PRJNA909223) and will be made publicly available before publication. The metabolomics data are available on MetaboLights with the unique identifier MTBLS6600 (www.ebi.ac.uk/metabolights/MTBLS6600). All supplemental, processed data tables are uploaded separately.

## Code availability

The code to perform the confounder and integrated statistical analyses are hosted at https://github.com/sxmorgan/pa-covid-multi-omics. Any further information required to reanalyze the data in this manuscript is available from the corresponding authors upon request.
