# Supplementary material for: Gut microbiota dysbiosis is associated with altered tryptophan metabolism and dysregulated inflammatory response in COVID-19
Source: NPJ Biofilms Microbiomes. 2024 Aug 1;10:66. doi: 10.1038/s41522-024-00538-0 (PMC11291933; doi:10.1038/s41522-024-00538-0)
Supplement: Supplementary file 1 — supplementary information [file 41522_2024_538_MOESM1_ESM.pdf]

Gut microbiota dysbiosis is associated with altered tryptophan metabolism and dysregulated inflammatory response in COVID-19

Supplemental Information

Supplementary Figure 1: Oropharyngeal microbiota differential abundance analysis and sequence data processing, related to Figure 2

Supplementary Figure 2: IFN response in COVID-19 patients, related to Figure 3

Supplementary Figure 3: Metabolite associations with disease severity at both early and late timepoints, related to Figure 4

Supplementary Table 1: Cohort characteristics, related to Table 1

Supplementary Table 2: Clinical lab parameters, related to Figure 1

Supplementary Table 3: Taxonomic profiles, related to Figures 2, Supplementary Figure 1

Supplementary Table 4: Immune response profiles, related to Figures 3, Supplementary Figure 2

Supplementary Table 5: Metabolite profiles, related to Figures 4, Supplementary Figure 3

Supplementary Table 6: Results from statistical analysis with clinical variables, related to Figures 2-4, Supplementary Figures 1-3

Supplementary Table 7: Integrated statistical analysis results, related to Figure 5

Supplementary Table 8: Quality Control reporting of metabolomics data according to mQACC standards

Supplementary Table 9: Ultra-high performance liquid chromatography (UHPLC) gradient for LC part

Supplementary Table 10: Ultra-high performance liquid chromatography (UHPLC) gradient for flow injection (FIA) part

Supplementary Table 11: Acquisition method parameters for liquid chromatography (LC) and flow injection analysis (FIA)

Supplementary Table 12: List of metabolite derivatives and their biological groups used for reference search

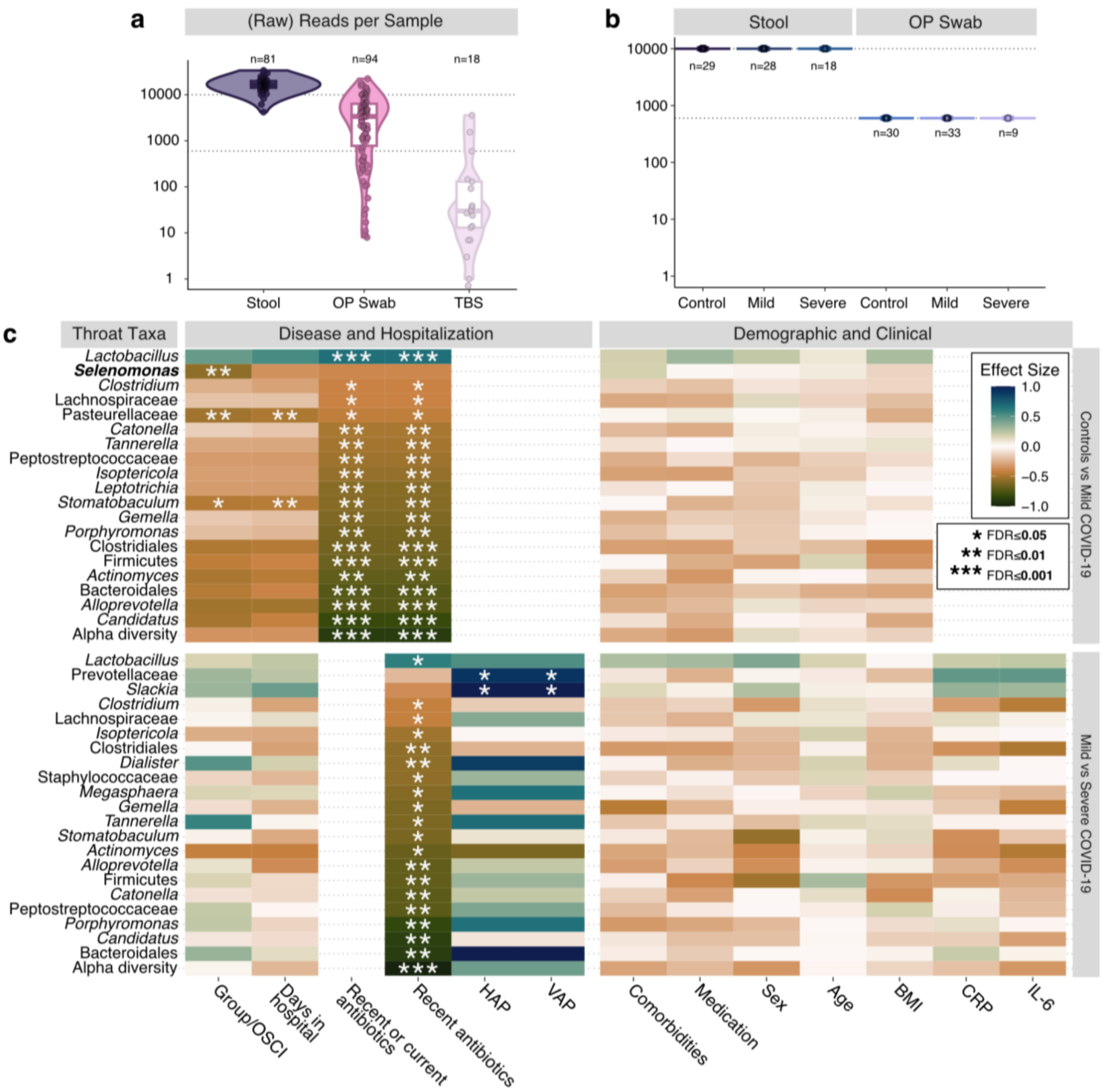

Supplementary Figure 1: Airway samples in COVID-19 patients had far fewer reads than stool samples, and were strongly sensitive to antibiotics. **a)** Raw (not rarefied) reads per sample, grouped by sample type. Grey dotted lines show selected rarefaction depths for stool and OP swabs, which resulted in removal of 6 and 22 samples, respectively. As only 5/18 TBS samples had more than 100 raw reads, all of these samples were excluded from further analysis. **b)** Rarefaction resulted in n=75 stool samples and n=72 OP swabs for downstream analysis (see main text **Fig. 2**). **c)** Subset of significant results from our differential abundance and confounder testing of the throat microbiota (see main text **Fig. 2c** caption, **Methods**, and **Table S6** for the full results). With the exception of P07 and P09, all mild COVID-19 patients received antibiotics shortly before intake, but not during their hospital stay (see main text **Fig. 1**), hence why the "recent" and "recent or current" antibiotics variables show practically identical signals (likewise, the same three patients had HAP/VAP). All but one patient with COVID-19 had recently received or was currently receiving antibiotics, making it impossible to distinguish mild from severe patients with respect to this variable. HAP, VAP, CRP, and IL-6 were either not relevant or not recorded for the control individuals. HAP: hospital-acquired pneumonia; VAP: ventilator-associated pneumonia; OSCI: ordinal scale for clinical improvement

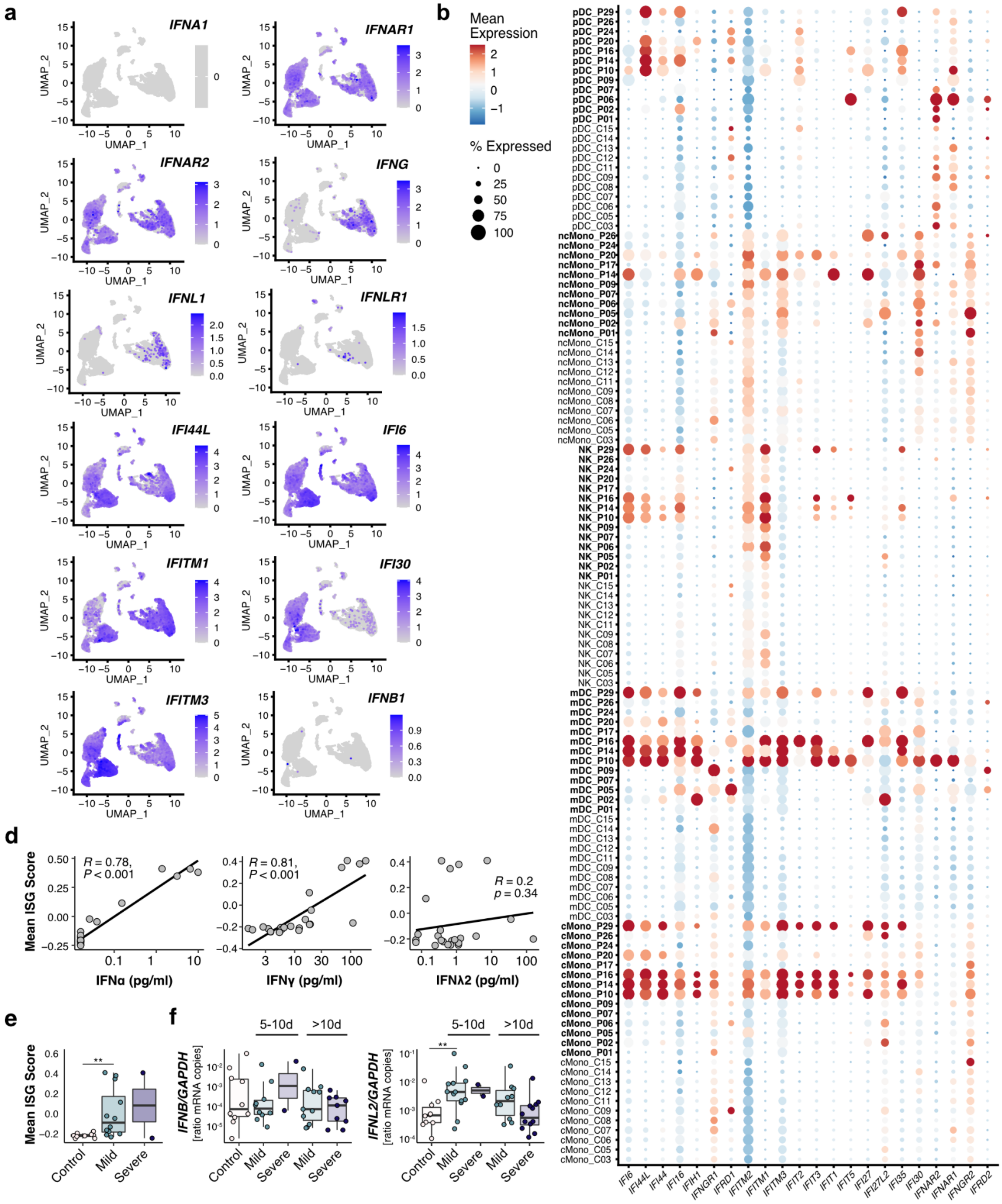

Supplementary Figure 2: IFN response in COVID-19 patients. a) Expression of IFN-related genes projected onto the UMAP of cell (sub)types of PBMCs identified by scRNAseq from 14 COVID-19 patients and 11 controls. **b)** Dot plots indicating expression of IFN-related genes in different cell types of PBMCs from patients and controls. **c)** Linear regression analysis of the ISG score (as shown in d versus levels of type I, II, and III IFN levels in plasma. **d)** ISG score (calculated by applying Seurat function *AddModuleScore* to IFN-stimulated genes) in PBMCs from controls and patients with mild and severe COVID-19. **e)** Relative oropharyngeal expression of type I and III IFNs in controls and patients with mild and severe COVID-19 at the early (<10 d after symptom onset) and late (>10 d after symptom onset) phase of infection. See also main text **Fig. 3.**

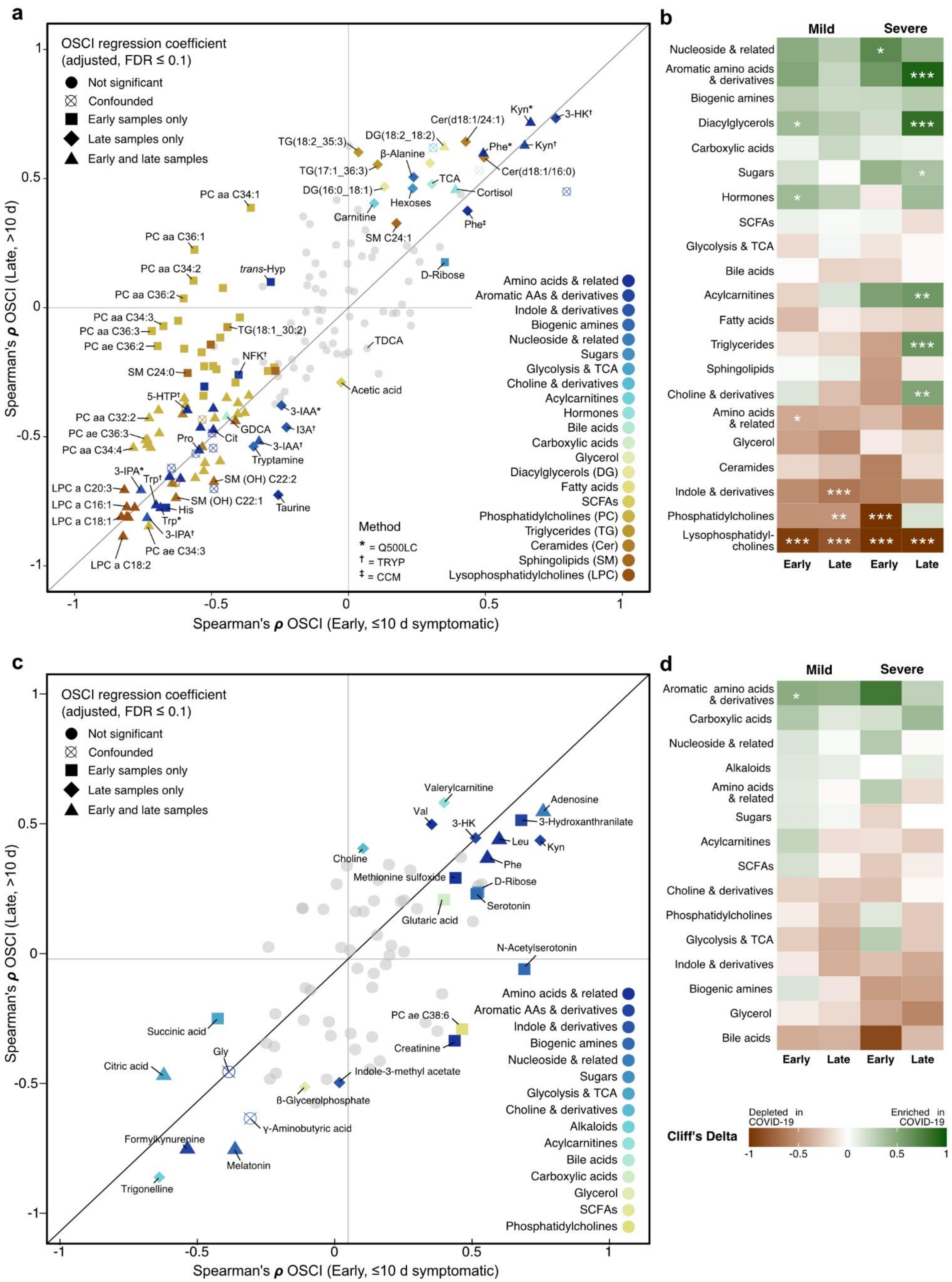
a
OSCI regression coefficient
(adjusted, FDR ≤ 0.1)
Not significant
Confounded
Early samples only
Late samples only
Early and late samples
Spearman's ρ OSCI (Late, >10 d)
Spearman's ρ OSCI (Early, ≤10 d symptomatic)
Kyn*
3-HK†
Cer(d18:1/24:1)
DG(18:2_18:2)
TG(18:2_35:3)
β-Alanine
Phe*
Kyn†
Cer(d18:1/16:0)
TG(17:1_36:3)
TCA
DG(16:0_18:1)
Cortisol
Hexoses
PC aa C34:1
Carnitine
Phe‡
SM C24:1
PC aa C36:1
PC aa C34:2
trans-Hyp
D-Ribose
PC aa C36:2
PC aa C34:3
TG(18:1_30:2)
PC aa C36:3
PC ae C36:2
TDCA
NFK†
SM C24:0
Acetic acid
5-HTP†
3-IAA*
PC aa C32:2
GDCA
I3A†
PC ae C36:3
Pro
Cit
PC aa C34:4
3-IAA†
Tryptamine
3-IPA*
Trp†
SM (OH) C22:2
LPC a C20:3
SM (OH) C22:1
LPC a C16:1
His
Taurine
Trp*
LPC a C18:1
3-IPA†
PC ae C34:3
LPC a C18:2
Method
* = Q500LC
† = TRYP
‡ = CCM
Amino acids & related
Aromatic AAs & derivatives
Indole & derivatives
Biogenic amines
Nucleoside & related
Sugars
Glycolysis & TCA
Choline & derivatives
Acylcarnitines
Hormones
Bile acids
Carboxylic acids
Glycerol
Diacylglycerols (DG)
Fatty acids
SCFAs
Phosphatidylcholines (PC)
Triglycerides (TG)
Ceramides (Cer)
Sphingolipids (SM)
Lysophosphatidylcholines (LPC)
b
Mild
Severe
Nucleoside & related
Aromatic amino acids & derivatives
Biogenic amines
Diacylglycerols
Carboxylic acids
Sugars
Hormones
SCFAs
Glycolysis & TCA
Bile acids
Acylcarnitines
Fatty acids
Triglycerides
Sphingolipids
Choline & derivatives
Amino acids & related
Glycerol
Ceramides
Indole & derivatives
Phosphatidylcholines
Lysophosphatidylcholines
Early
Late
Early
Late
c
OSCI regression coefficient
(adjusted, FDR ≤ 0.1)
Not significant
Confounded
Early samples only
Late samples only
Early and late samples
Spearman's ρ OSCI (Late, >10 d)
Spearman's ρ OSCI (Early, ≤10 d symptomatic)
Valerylcarnitine
Adenosine
Val
3-HK
3-Hydroxanthranilate
Leu
Kyn
Choline
Phe
Methionine sulfoxide
D-Ribose
Serotonin
Glutaric acid
N-Acetylserotonin
PC ae C38:6
Succinic acid
Creatinine
Gly
Citric acid
Indole-3-methyl acetate
β-Glycerolphosphate
γ-Aminobutyric acid
Formylkynurenine
Melatonin
Trigonelline
Amino acids & related
Aromatic AAs & derivatives
Indole & derivatives
Biogenic amines
Nucleoside & related
Sugars
Glycolysis & TCA
Choline & derivatives
Alkaloids
Acylcarnitines
Bile acids
Carboxylic acids
Glycerol
SCFAs
Phosphatidylcholines
d
Mild
Severe
Aromatic amino acids & derivatives
Carboxylic acids
Nucleoside & related
Alkaloids
Amino acids & related
Sugars
Acylcarnitines
SCFAs
Choline & derivatives
Phosphatidylcholines
Glycolysis & TCA
Indole & derivatives
Biogenic amines
Glycerol
Bile acids
Early
Late
Early
Late
Cliff's Delta
Depleted in COVID-19
Enriched in COVID-19
-1
-0.5
0
0.5
1

Supplementary Figure 3: Metabolite associations with disease severity at both early and late timepoints. a) Spearman correlations between plasma metabolite concentrations and OSCI scores at the time of sampling were calculated using either early (n=26) or late (n=40) subsets of COVID-19 patient samples and healthy controls (n=30), and are shown contrasted with one another. Metabolites are annotated to show their biochemical class and analytical method. Shapes were assigned according to the integrated output from our modeling with clinical variables in each subset (early and late) separately, after adjusting for case-control disease status. Compounds closer to the diagonal line displayed consistent severity correlations regardless of sample timepoint, while levels of several phosphatidylcholines were negatively correlated with disease severity in early samples only. **b)** Cliff's delta effect sizes for different subsets of COVID-19 patients compared to controls for each chemical class. Adjusted significances from Mann-Whitney U tests which were not confounded from our modeling analysis with clinical covariates are depicted (FDR<0.1=*, FDR<0.01=**, FDR<0.001=***). **c-d)** The same as in a and b except for urine metabolites. See also main text Fig. 4. Phe: phenylalanine, Pro: proline, Cit: citrullin, *trans*-Hyp: *trans*-4-hydroxyproline, Trp: tryptophan, Kyn: kynurenine, 5-HTP: 5-hydroxytryptophan, 3-HK: 3-hydroxykynurenine, NFK: N-Formylkynurenine, TDCA: taurodeoxycholic acid, GDCA: glycodeoxycholic acid, TCA: taurocholic acid, 3-IPA: indole-3-propionic acid, 3-IAA: indole-3-acetic acid, I3A: indole-3-carboxaldehyde; HAP: hospital-acquired pneumonia

The following tables are currently available at www.github.com/sxmorgan/pa-covid-multi-omics.

Supplementary Table 1 (S1): Cohort characteristics, related to Table 1

Supplementary Table 2 (S2): Clinical lab parameters, related to Figure 1

Supplementary Table 3 (S3): Taxonomic profiles, related to Figure 2

Supplementary Table 4 (S4): Immune response profiles, related to Figures 3, Supplementary Figure 2

Supplementary Table 5 (S5): Metabolite profiles, related to Figures 4, Supplementary Figure 3

Supplementary Table 6 (S6): Results from statistical analysis with clinical variables, related to Figures 2-4, Supplementary Figures 1 and 3

Supplementary Table 7 (S7): Integrated statistical analysis results, related to Figure 5

Supplementary Table 8 (S8): Quality Control reporting of metabolomics data according to mQACC standards

Supplementary Table 9: Ultra-high performance liquid chromatography (UHPLC) gradient for LC part

	**LC1**				**LC2**			
No	Time [min]	Flow [mL/min]	A [%]	B [%]	Time [min]	Flow [mL/min]	A [%]	B [%]
1	0.00	0.8	100	0	0.00	0.8	100	0
2	0.25	0.8	100	0	0.25	0.8	100	0
3	1.50	0.8	88	12	0.50	0.8	75	25
4	2.70	0.8	82.5	17.5	2.00	0.8	50	50
5	4.00	0.8	50	50	3.00	0.8	25	75
6	4.50	0.8	0	100	3.50	0.8	0	100
7	4.70	1.0	0	100	4.70	1.0	0	100
8	5.00	1.0	0	100	5.00	1.0	0	100
9	5.10	1.0	100	0	5.10	1.0	100	0
10	5.80	1.0	100	0	5.80	1.0	100	0

Supplementary Table 10: Ultra-high performance liquid chromatography (UHPLC) gradient for flow injection (FIA) part

No	Time [min]	Flow [mL/min]	A [%]	B [%]
1	0.0	0.03	0	100
2	1.6	0.03	0	100
3	2.4	0.20	0	100
4	2.8	0.20	0	100
5	3.0	0.03	0	100

Supplementary Table 11: Acquisition method parameters for liquid chromatography (LC) and flow injection analysis (FIA)

Option	Parameter	LC1	LC2	FIA1	FIA2
MS	Scan type	MRM	MRM	MRM	MRM
	Polarity	Positive	Negative	Positive	Negative
	MRM detection window (sec)	30	30	-	-
	Duration (min)	5.45	5.45	2.95	2.95
	Delay time (sec)	0	0	0	0
	Cycle (sec)	0.25	0.15	N/A	N/A
Advanced MS	Resolution Q1	Unit	Unit	Unit	Unit
	Resolution Q3	Unit	Unit	Unit	Unit
	Intensity threshold	0	0	0	0
	Setting time (ms)	0	0	0	0
	Pause between mass ranges (ms)	2	2	5.007	3
Source/ gas	Curtain gas	45	20	20	10
	Collision gas	9	8	9	9
	Ion spray voltage	5500	-4500	5500	5500
	Temperature	500	650	200	350
	Ion source gas 1	60	40	40	30
	Ion source gas 2	70	40	50	85

MS: mass spectrometry

Supplementary Table 12: List of metabolite derivatives and their biological groups used for reference search

Group	Metabolite	Abbreviation	Detected as
AA	Alanine	Ala	3TMS
			2TMS
AA	Asparagine	Asn	2TMS
AA	Aspartic acid	Asp	2TMS
			3TMS
AA	Cysteine	Cys	3TMS
AA	Glycine	Gly	2TMS
			3TMS
AA	Isoleucine	Ile	1TMS
			2TMS
AA	Leucine	Leu	1TMS
			2TMS
AA	Lysine	Lys	3TMS
AA	Methionine	Met	1TMS
			2TMS
AA	Phenylalanine	Phe	1TMS
			2TMS
AA	Proline	Pro	1TMS
			2TMS
AA	Serine	Ser	2TMS
			3TMS
			4TMS
AA	Threonine	Thr	2TMS
			3TMS
AA	Tryptophan	Trp	2TMS
AA	Tyrosine	Tyr	3TMS
AA	Valine	Val	1TMS
			2TMS

Glycerol	Dihydroxyacetone phosphate	DHAP	1MeOX 3TMS
Glycerol	Glycerol	Glyc	3TMS
Glycerol	Glycerol-3-phosphate	Glyc3P	4TMS
Glycolysis	Fructose-6-phosphate	F6P	1MeOX 6TMS
Glycolysis	Glucose-6-phosphate	G6P	1MeOX 6TMS
Glycolysis	Glyceric acid-3-phosphate	GA3P	4TMS
Glycolysis	Lactic acid	Lac	2TMS
Glycolysis	Phosphoenolpyruvic acid	PEP	3TMS
Glycolysis	Pyruvic acid	Pyr	1MeOX 1TMS
Nucleobase	Adenine	Adenine	2TMS
Nucleobase	Uracil	Uracil	2TMS
Nucleoside	Adenosine	Adenosine	3TMS
			4TMS
Nucleoside	Cytosine	Cytosine	2TMS
Others	Butanoic acid, 3-hydroxy-	But3h	2TMS
Others	Butanoic acid, 4-amino-	But4am	3TMS
Others	Erythritol	Ery	4TMS
Others	Glutaric acid	Glut	2TMS
Others	Glyceric acid	Glyc	3TMS
PPP	Ribose-5-phosphate	R5P	1MeOX 5TMS
PPP	Ribose	Ribose	1MeOX 4TMS
TCA	Citric acid	Cit	4TMS
TCA	Fumaric acid	Fum	2TMS
TCA	Glutaric acid, 2-hydroxy-	2HG	3TMS
TCA	Glutaric acid, 2-oxo-	aKG	1MeOX 2TMS
TCA	Malic acid	Mal	3TMS
TCA	Succinic acid	Suc	2TMS

AA: Amino acids. PPP: Pentose phosphate pathway. TCA: Tricarboxylic acid cycle. TMS: Trimethylsilyl derivatives. MeOX: Methoxyamine hydrochloride
